# Comprehension through explanation as the interaction of the brain’s coherence and cognitive control networks

**DOI:** 10.3389/fnhum.2015.00562

**Published:** 2015-10-23

**Authors:** Jarrod Moss, Christian D. Schunn

**Affiliations:** ^1^Department of Psychology, Mississippi State UniversityMississippi State, MS, USA; ^2^Learning Research and Development Center, University of PittsburghPittsburgh, PA, USA

**Keywords:** comprehension, reading, reading strategy, cognitive control, coherence, psychophysiological interaction

## Abstract

Discourse comprehension processes attempt to produce an elaborate and well-connected representation in the reader’s mind. A common network of regions including the angular gyrus, posterior cingulate, and dorsal frontal cortex appears to be involved in constructing coherent representations in a variety of tasks including social cognition tasks, narrative comprehension, and expository text comprehension. Reading strategies that require the construction of explicit inferences are used in the present research to examine how this coherence network interacts with other brain regions. A psychophysiological interaction analysis was used to examine regions showing changed functional connectivity with this coherence network when participants were engaged in either a non-inferencing reading strategy, paraphrasing, or a strategy requiring coherence-building inferences, self-explanation. Results of the analysis show that the coherence network increases in functional connectivity with a cognitive control network that may be specialized for the manipulation of semantic representations and the construction of new relations among these representations.

## Introduction

Reading a textbook or other expository text is a common task performed by students on a routine basis. However, reading requires the construction of an elaborate and well-connected web of knowledge in the reader’s mind. Well-connected, or coherent, representations are essential for initial text comprehension, retention of knowledge, and the ability to use that knowledge in later applications (Chi et al., 1994; McNamara et al., 1996; McNamara and Kintsch, 1996). These representations that result from comprehension are termed situation models in theories of discourse comprehension (Zwaan et al., 1995; Kintsch, 1998). Constructing a coherent situation model requires a number of processes including propositionalizing the text, activating relevant long-term knowledge, and constructing inferences about implied relationships in the text (Kintsch, 1998). The networks of brain regions that accomplish these tasks are beginning to be identified, but the mapping of these networks onto cognitive theories of comprehension is still an active area of research.

### Coherence Building Regions and Expository Text

Constructing coherent representations applies not only to reading comprehension but also to how people think about the actions of other people. A set of common brain regions appears to be active in social cognition (Mar, 2011; Spunt and Lieberman, 2012) and reading narrative texts (Ferstl et al., 2008). These regions include the dorsomedial prefrontal cortex (dmPFC), posterior cingulate cortex (PCC), the precuneus, the inferior frontal gyrus (IFG), and the angular gyrus. Research from social cognition has termed this set of regions a “mentalizing network” (Mar, 2011). This mentalizing network is thought to be common to social cognition and narrative comprehension because both domains involve theory-of-mind as a significant component process in which people simulate the thoughts and beliefs of other people (or characters). However, all of these regions of the mentalizing network except the dmPFC are also seen when comprehending expository science texts (Moss et al., 2011, 2013). It is difficult to understand how reading an expository text about cell mitosis engages a reader’s theory-of-mind processes. However, all three of these domains do require the construction of coherent situation models, which involves making inferences and retrieval of relevant knowledge.

The brain regions involved in coherence-building discourse processes have been examined in various ways including the comparison of incoherent and coherent sets of sentences (e.g., Ferstl and von Cramon, 2001, 2002), single sentences compared to narratives composed of multiple related sentences (e.g., Xu et al., 2005), or sets of sentences which require predictive inferences compared to those that do not (Mason and Just, 2011). However, with the exception of a couple of studies (Moss et al., 2011, 2013) most of these studies have examined coherence building only in narrative text. Narrative text is likely to engage theory-of-mind processes as part of the coherence-building process, making it difficult to determine whether these brain regions are specific to theory-of-mind or contribute to coherence building in general. The fact that expository text comprehension activates a similar network including the angular gyrus, precuneus, dorsal superior frontal gyrus (dSFG), and PCC leads to the hypothesis that these regions form a coherence-building network independent from theory-of-mind processes (Moss et al., 2011, 2013). Discourse comprehension involves a number of processes including the controlled allocation of attention to retrieve and manipulate structured representations in working memory that came from propositionalizing the text and information retrieved from long-term memory. This coherence-building network likely includes regions that accomplish these functions, but there has been little agreement on a function-to-region mapping in the literature. However, a number of studies strongly link the dmPFC region to theory-of-mind (Fletcher et al., 1995; Ferstl and von Cramon, 2002; Mar, 2011; Mason and Just, 2011), and this region was not identified as being active for expository text (Moss et al., 2011, 2013). Therefore, the use of expository text seems a promising method for exploring the neural regions involved in coherence building separate from theory-of-mind, and methods examining structural and functional connectivity could potentially further elucidate the mapping of brain regions and networks to the component cognitive processes theorized to underlie discourse comprehension.

### Self-Explanation Strategies to Build Coherence in Expository Text Reading

In addition to these reasons for examining expository text, it is also the case that expository text is often missing many of the inter-sentence links that are necessary for building a coherent representation during comprehension (i.e., it is low in cohesion; Duran et al., 2007). In response, readers have to engage in many bridging inferences as well as elaborative inferences using their prior knowledge (McNamara and Kintsch, 1996; McNamara, 2004). Therefore, expository text more strongly elicits coherence-building activity than does narrative text. Unfortunately, while inferences during narrative text are often made automatically because of the relevant semantic knowledge they activate, low-domain-knowledge readers often have difficulty making the necessary inferences for low-cohesion expository text (McNamara et al., 1996). This limitation has been overcome in prior work and the work reported here by training readers to use a self-explanation reading strategy that involves the use of multiple types of inferences for coherence building (Moss et al., 2011, 2013).

Self-explanation is a reading strategy that focuses on the coherence-building processes of inferencing and elaboration, and it has been shown to be effective at improving readers’ comprehension when students are trained or prompted to use it (Chi et al., 1994; McNamara, 2004). Training readers to self-explain is done through focused practice with the five component elements of self-explanation: comprehension monitoring, paraphrasing, elaboration, bridging, and prediction. Comprehension monitoring is being aware of whether the text is being successfully understood while reading. Paraphrasing is putting the text into one’s own words in order to help activate relevant semantic knowledge in long-term memory and prepare the reader to make further inferences. Inferences are necessary in text comprehension because texts do not explicitly state all relevant pieces of information explicitly (Kintsch, 1998). Elaboration involves making inferences that aid in understanding the current text by using knowledge from memory (i.e., semantic knowledge about the world or prior experiences with related content). Bridging involves making inferences that aid in understanding the current text by drawing on information from prior sentences in the text. Prediction involves making a forward inference at the end of a sentence or paragraph about what information will be contained in the next section of the text. Self-explanation brings these elements together so that readers explain the text to themselves by putting the text into their own words, making elaborative inferences based on their prior knowledge, and making bridging inference across sentences and paragraphs of the text about ambiguous or confusing aspects of the text. These elaborative and bridging inferences are thought to help to build coherence in a reader’s representation by forming connections between propositions in their mental representation of the text, or situation model, that would not have been formed automatically (Kintsch, 1998; McNamara, 2004). Other less effective strategies that readers could employ are rereading the text or only paraphrasing the text by putting the text into their own words (Chi et al., 1994; McNamara, 2004; Moss et al., 2011).

The current study uses this expository-text reading-strategy paradigm to investigate the brain’s coherence building network. This paradigm is robust and by contrasting conditions in which participants use self-explanation with conditions in which they only paraphrase, one can isolate activity related to coherence-building inferences that is highly replicable (Moss et al., 2013). Paraphrasing was initially selected as a good strategy for contrast with self-explanation because it is a component strategy of self-explanation, but it does not involve making inferences. Therefore, in the initial work examining strategic reading comprehension, paraphrasing activity could be subtracted from self-explanation activity to isolate the inference-producing components of self-explanation (Moss et al., 2011). In addition, paraphrasing has continued to be used as a good condition for contrast with self-explanation because it activates cognitive control regions to a similar degree as self-explanation (Moss et al., 2011, 2013). However, this prior work has not examined measures of functional connectivity that might lead to a better characterization of the way in which this coherence network interacts with other regions or networks of regions in the brain.

### Functional Connectivity Methods for Examining Coherence Processes

There are many methods of examining connectivity in the brain including the use of diffusion tensor imaging (DTI) to examine anatomical connectivity as well as multiple measures that use electrophysiological and functional imaging data to examine functional and effective connectivity. Many of these methods have been used to examine language comprehension. For example, resting-state functional magnetic resonance imaging (rs-fMRI) and DTI have been combined to examine connectivity of regions activated by word and sentence reading (Turken and Dronkers, 2011). DTI and measures of functional connectivity related to Grainger causality have also been used to examine sentence comprehension (Saur et al., 2010). However, discourse-level processing is known to engage additional neurocognitive processes beyond that of sentence-level processing (Xu et al., 2005; Yarkoni et al., 2008).

Discourse-level studies have examined how reading narrative text with the goal of making predictive inferences changes effective connectivity between language-processing regions using dynamic causal modeling (DCM; Chow et al., 2008). In addition, other functional connectivity measures including spatial independent component analysis, rs-fMRI, psychophysiological interaction (PPI), and other correlation techniques have been used to examine narrative comprehension (Mason and Just, 2011; Spotorno et al., 2012; Berns et al., 2013; Smallwood et al., 2013; AbdulSabur et al., 2014; Smirnov et al., 2014). From these studies, it is clear that coherence building regions and theory-of-mind regions identified from analyses focusing on increases in activity also interact with other brain regions via increases in functional connectivity.

Prior connectivity studies provide a basis for hypothesizing that the coherence-building processes observed in the angular gyrus, PCC, and dSFG in expository text comprehension also engage networks of regions involving the controlled retrieval and selection of representations from semantic memory. For example, when individuals read with the goal of making predictive inferences, connectivity between regions of the posterior temporal cortex increases with regions of the IFG and anterior prefrontal cortex associated with semantic memory retrieval, coherences assessment, and control of attention (Chow et al., 2008). Using spatial independent component analysis, language-processing regions in the temporal cortex were found to show connectivity with “mentalizing” regions including the dmPFC as well as with regions of the IFG and supplementary motor area (SMA) linked to semantic memory and the processing of sequential relationships respectively (AbdulSabur et al., 2014). Repeated rs-fMRI scans over multiple days of reading a novel led to increased hub-like connectivity of the angular gyrus (Berns et al., 2013). Another narrative study used PPI and found increased connectivity between dmPFC and IFG when participants read ironic passages as compared to literal passages (Spotorno et al., 2012). These studies show that the brain’s coherence network is engaging additional regions and networks that involve cognitive control to retrieve and select representations from semantic memory. However, the function of each of the coherence-building regions has not been clearly specified or related to component processes of cognitive theories of comprehension.

There have been attempts to explain the relationship between the recruited networks and regions of the coherence-building network such as the angular gyrus. For example, increases in connectivity between frontal regions and regions of the posterior temporal cortex, temporal parietal junction, and the angular gyrus have been hypothesized to be involved in integrating a sentence with discourse context including the controlled retrieval and selection of relevant semantic information (Saur et al., 2010). Individual differences in resting state connectivity between the PCC and angular gyrus have been associated with more task focus and fewer task unrelated thoughts while reading (Smallwood et al., 2013) indicating that these regions might be engaged in some form of attentional control. A study using PPI with either pictures matching or not matching a spoken narrative found unique patterns of increased connectivity with regions of the coherence-building network and the IFG (Smirnov et al., 2014). In that study, the angular gyrus and IFG BA 44 and 45 showed increased connectivity, while the PCC showed increased connectivity only with BA 45. These patterns of connectivity indicate that the angular gyrus may be more involved in the retrieval or use of semantic memories associated with BA 44 than the PCC.

These kinds of functional connectivity analyses seem a promising source of information to tease apart the functions performed by these regions because they provide additional information going beyond which regions were more active during a particular task. This advantage especially holds for this network because so many of the coherence-building regions are all active during the same tasks, including while at rest (i.e., the default mode network). Therefore, the current paper focuses on a functional connectivity analysis of an expository discourse comprehension task.

### Current Study

The current paper reports the results of a PPI analysis examining the increase in functional connectivity between these coherence-building regions of interest (ROIs) and other brain regions. PPI was selected as the analysis method because it identifies regions that increase in connectivity across two different psychological conditions (Friston et al., 1997; O’Reilly et al., 2012). A limitation of PPI is that it is a method with relatively low statistical power (O’Reilly et al., 2012). The use of a common paradigm across three prior studies using strategic reading of expository texts therefore provides a unique opportunity to examine changes in connectivity associated with coherence building without being confounded by lower-level language processes or theory-of-mind processes. Lower-level language processes are subtracted out in these studies because the contrast of self-explanation with paraphrasing means that all common processes are removed (i.e., text perception, text propositionalization, speech planning). In addition, prior analysis of the utterances used by participants in self-explanation and paraphrasing find them to be equivalent in syntactic complexity (Moss et al., 2011, 2013). Two of these strategic reading comprehension datasets come from prior publications focused on identifying cognitive control and discourse-processing regions involved in strategic reading (Moss et al., 2011) and identifying correlates of mind wandering during strategic reading (Moss et al., 2013). The third set of data comes from a currently unpublished study examining metacognitive monitoring of comprehension. In both of the previously published studies, only differences in peak activation were examined and not functional connectivity.

The three ROIs shown in Table 1 were used as seed regions in the PPI analysis because they were identified from a conjunction analysis across multiple strategic reading studies as regions consistently showing increased activity during self-explanation when compared to paraphrasing a text (Moss et al., 2013). These ROIs included regions of the angular gyrus, PCC, and dSFG. This PPI analysis should provide insight into the way in which these regions, referred to as a coherence-building network, recruit additional networks of regions in service of making coherence-related inferences necessary for successful text comprehension.

**Table 1:** **ROIs for PPI analysis that have been found to activate more for self-explanation than for paraphrasing in a conjunction analysis (Moss et al., 2013)**.

ROI label	Center of mass talairach coordinates	Brodmann areas	Size (mm^3^)
L Angular g	(−45, −64, 31)	39	1,782
L dSFG	(−18, 29, 50)	8	243
L PCC	(−4, −34, 36)	23, 31	648

In addition to the PPI analysis, two other sets of analyses were conducted to further examine which regions are involved in strategic discourse comprehension. First, activation for self-explanation and paraphrasing were compared to see if additional regions with smaller effect sizes show significant differences using the combined data analyzed in this paper. It is possible that these additional regions were not previously identified for lack of power within each of the individual studies. Second, in order to provide additional insight into the function and connectivity of regions identified in the PPI analyses, resting state connectivity data from the Human Connectome Project (Van Essen et al., 2012, 2013; Smith et al., 2013) was used to get a qualitative sense of the brain networks involved in coherence building during self-explanation.

## Materials and Methods

### Participants

Data from a total of 58 right-handed, native English speakers were included in the analyses (35 female, *M* age = 20.7 years, range = 18–28). Thirty-six of these participants were recruited from the University of Pittsburgh and Carnegie Mellon University communities as part of previously published studies (15 from Moss et al., 2013; and 21 from Moss et al., 2011). The remaining 22 participants were recruited from Mississippi State University. All participants were paid for their participation. The University of Pittsburgh Institutional Review Board approved data collection procedures at the University of Pittsburgh and the Mississippi State University Institutional Review Board approved data collection procedures at Mississippi State University. All relevant national and institutional regulatory standards were followed and all participants provided informed consent prior to participation.

### Materials

Further details of the materials for two of the studies have been reported elsewhere (Moss et al., 2011, 2013). Six texts were used across the three studies. In one of the studies (Moss et al., 2013), three 15-paragraph introductory physics texts were used on the topics of pulleys, electricity, and forces, with most paragraphs containing an accompanying diagram depicting the topic discussed in that paragraph. The other two studies used three 12-paragraph introductory biology texts on the topics of cell mitosis, DNA structure, and heat exchange in the circulatory system.

### Design

We combine data from three studies that had a very similar design intended to examine the neural correlates of different reading strategies. Two of the studies compared three reading strategies: rereading, paraphrasing, and self-explaining (Moss et al., 2011, 2013). The third study only compared the paraphrasing and self-explanation strategies. The design common to all three studies will be discussed here along with the details of the third unpublished study. Particular details of the design of the other two studies not relevant to the current paper can be found in the corresponding papers. Unless otherwise mentioned, description of the design and procedure is common to all three studies.

Reading strategy was manipulated within-subjects such that each participant performed all reading strategies. Each participant was instructed to use a given reading strategy to read all of a given text. The assignment of reading strategies to texts was counterbalanced across participants. The order in which participants performed the reading strategies was randomized.

Each multi-paragraph text was divided into three parts each consisting of an equal number of paragraphs (i.e., 12 paragraphs broken up into three sections of four paragraphs each). Each of these multi-paragraph sections was presented in a single data acquisition run (for a total of nine runs). Because strategies were assigned to texts, participants were always performing a single strategy during each acquisition run. A data acquisition run for the first section for each of the texts was run before the data acquisition run for the second section of each text and so on. For example, this organization implies that the first and second sections of a particular text were separated by a section of each of the other two texts (e.g., Text1-Section1, Text2-Section1, Text3-Section1, Text1-Section2, Text2-Section2, …). The sections were presented in this fashion so that each reading strategy would be performed once in each third of the acquisition session in order to help control for potential confounding effects (e.g., fatigue). The only difference in this design across studies is that the unpublished study, containing only self-explanation and paraphrasing, only had two reading strategies and therefore only six data acquisition runs (e.g., Text1-Section1, Text2-Section1, Text1-Section2, …).

### Procedure

**Figure 1:**
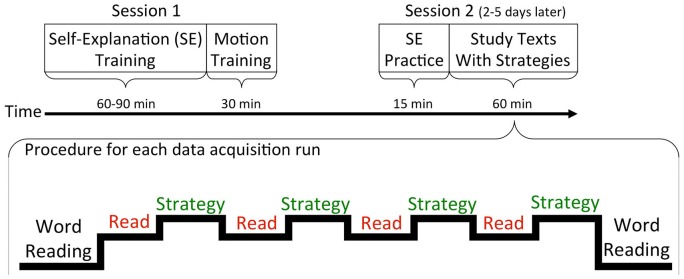
**Procedure for the two sessions is shown at the top.** At the bottom is a depiction of the sequence of tasks within one fMRI data acquisition run. The beginning and ending of each run either included a word-reading task (in one study) or a fixation task (in two studies).

Data collection for all of the studies took place over two sessions, with fMRI data collected only during the second session. The procedure for the unpublished study will be described here in detail. The procedure for the other two studies is very similar, with relevant differences mentioned as appropriate. See Figure 1 for a depiction of each session’s structure.

#### Session 1

Participants completed self-explanation training based on Self-Explanation Reading Training (SERT) as descried by McNamara (2004). For SERT, participants were presented, via a simple slideshow with audio, with examples of someone performing each of the five self-explanation elements: paraphrasing, bridging, elaboration, comprehension monitoring and prediction. Following these examples, participants were given practice performing each of these explanations while being monitored by the experimenter. The experimenter encouraged the participant to try again if the participant failed to give a good example of each of the strategies. Following this training on individual self-explanation elements, participants practiced self-explaining two texts using all five elements. The experimenter monitored performance during this practice and provided encouragement to utilize all five self-explanation elements if participants were not using one or more of the SERT strategies. The SERT training took about 1 h. In the two published studies, this training session was identical except that an intelligent tutoring system, iSTART, was used to train participants for 90 min (McNamara et al., 2004; Levinstein et al., 2007). iSTART is based on SERT with the primary difference being that a computer tutor provides the feedback instead of the human experimenter. iSTART’s interface and instruction is oriented toward younger populations using simpler texts and takes a bit longer than SERT training, so it was replaced by a human tutor in the unpublished study. All training was done with practice texts that were of a similar expository nature but contained different content than the texts in the second session.

After SERT training, the participants were provided with task practice in an MRI simulator. The MRI simulator was designed to closely simulate the physical conditions of the MRI scanner and included a magnetic head tracking system to present feedback to the participant regarding head movement. The simulator practice was done to screen for claustrophobia, to train participants to perform the talk aloud task without excessive head motion, and to provide them with practice on the experimental task using the same button response system they would use during the scanning session. In the simulator, participants were presented with two practice texts that were of a similar expository nature but contained different content than the texts in the experiment. Before each block of paragraphs, instructions were presented on the screen indicating the reading strategy to use for that block.

During the practice session and the MRI session, the procedure for reading and performing the strategy on each text was as follows. The title of the text was centered on the top of the screen with the paragraph appearing on the center of the screen. Along the bottom of the screen was a prompt reminding the participant of the current strategy. Participants were instructed to read the paragraph aloud once, and then to press a button on a response glove. Once they did so, the color of the paragraph’s text changed from black to blue which served as a cue that they were to perform the given reading strategy aloud. The participants then paraphrased or self-explained the text and pressed a button (rereading was used as an additional reading strategy in the prior two studies). Initial reading and performing the given reading strategy were each self-paced subject to the constraint that they each could take no longer than 45 s, at which point the presentation program behaved as if a button were pressed. After participants completed the learning strategy for a particular paragraph, they were presented with prompts to assess how often they were mind wandering and their metacognitive assessment of their comprehension. The mind wandering prompt was also used in one of the prior studies. None of these prompts is the focus of the current paper.

For self-explanation, participants were encouraged by the instructions to use all five self-explanation elements. In the paraphrase condition, participants were told to put each sentence in the paragraph into their own words without using any of the other SERT strategies.

#### Session 2

This session began with a 15 min self-explanation practice session for additional practice self-explaining. fMRI data was collected for the remainder of the session. All tasks were presented using E-Prime (Schneider et al., 2002). To be able to verify strategy use within each condition during scanning, verbal responses were collected using the Persaio active noise canceling microphone system (Psychology Software Tools, Inc., Pittsburgh, PA, USA), which almost entirely removed the scanner background noise.

Following localizer and high-resolution anatomical T1-weighted scans, participants began the task of reading and using the reading strategies on two biology texts across six data acquisition runs. Each 12-paragraph text was broken up into three sections of four paragraphs each and one section of paragraphs was presented during each run (see Figure 1). The only difference from the MRI simulator trial procedure was that a 30 s task was placed before and after each section of paragraphs. In the unpublished study, a word reading task was used that contained words from a third biology text but the words were presented in random order such that there was no sentence or higher-level discourse structure. This task was included to serve as a control to compare against reading the actual paragraphs, but it is not the focus of the current paper. In the prior two published studies, in place of this word-reading task, a 30 s rest period (i.e., a fixation cross) was placed before and after each block of paragraphs.

### MRI Data Acquisition and Analysis

Structural and functional images for the unpublished study were collected on a whole-body GE 3T scanner at Mississippi State University. The scanning session began with the acquisition of structural images, which included scanner-specific localizers and volume anatomical series. The volume anatomical scan was acquired in a sagittal plane (1 mm^3^) using the GE SPGR sequence and the functional data were co-registered to these images. The functional runs were acquired as 28 axial slices using a T2*-weighted echo-planar imaging (EPI) pulse sequence (TE = 30 ms, TR = 2000 ms, FOV = 24 cm, slice thickness = 4 mm with no gap, flip angle = 76, in-plane resolution = 3.75 × 3.75 mm). A similar fMRI acquisition was done for the prior two published studies using a Siemens 3T Trio scanner at the University of Pittsburgh (for detailed scan parameters see Moss et al., 2011, 2013).

#### Preprocessing

The raw neuroimaging data were preprocessed and analyzed using the AFNI software package (Cox, 1996). Preprocessing included high-pass filtering, slice scan time correction, three-dimensional motion correction, and spatial smoothing. All functional images were realigned to the first image of each run, which were aligned to the first run of each subject. Estimated motion was examined in order to exclude time points during which excessive motion occurred from statistical analysis. Overall, the training in the MRI simulator in session 1 was successful at helping to minimize motion. Peak displacement and rotation within a data collection run were less than 2 mm or 2 degrees in all directions except for inferior-superior displacement and pitch rotation, and in these directions motion was less than 3 mm or 3 degrees. Only one participant from one of the prior studies had excessive head motion (Moss et al., 2011), and that participant was not included in prior work or in the participant count in this paper. Prior research has also shown that artifacts due to speech are minimized by comparing two conditions that both involve overt speech as is the case with the paraphrase and self-explanations contrast being examined in this paper (Barch et al., 1999). These are peak values, and motion was generally much less than these peak values. Combining the six estimated motion parameters yields an average root mean square of 0.15 for participants with a range of 0.07–0.38. Using the measure of framewise displacement (FD; Power et al., 2012), average estimated motion was 0.29 mm with individual participant averages ranging rom 0.12–0.51 mm. Images with a large number of outliers as determined by the AFNI program 3dToutcount were visually inspected and censored from statistical analyses if the images were obviously distorted. As an additional step, an FD threshold of 0.7 mm was used to automatically censor or “scrub” data that exceeded the threshold. In all cases, significant clusters found using the outlier method were also identified using the FD threshold. Some individual clusters varied slightly in size between the two methods, but neither method produced systematically smaller or larger clusters. Given the similarity in results, it seems unlikely that head motion biased the results, and, for simplicity, only the results with the outlier method are reported here.

The signal for each voxel was spatially smoothed (7 mm FWHM). Each participant’s anatomical images were co-registered to their functional images by applying a transformation to the anatomical images. The structural and functional images were then transformed using an affine transformation using AFNI’s @auto_tlrc command into a canonical Talairach space with 3 mm isometric voxels (Talairach and Tournoux, 1988). Prior to analyzing the data with a voxel-wise general linear model, the probabilistic ICA method MELODIC from the FSL software package (Beckmann and Smith, 2004) was used to reduce noise, following Smith et al. (2010). In particular, the time course for each component was correlated with motion to determine the top 10% components to remove based on motion. High frequency noise components were eliminated by rank ordering the components by the frequency at which their power was highest (using a Fourier transform) and removing the top 10% of these components. Finally, the top 10% of components showing the greatest TR-to-TR signal change (i.e., a discontinuous spike) were also removed. Comparison of analyses with and without this noise reduction step did not differ greatly, but this step did increase the peak *t*-statistics for most clusters. The final preprocessing step was to scale each run by its mean so that all runs had a mean signal of 100 so that beta coefficients of regressors scaled to a −1 to 1 range can be interpreted as percent signal change.

Analyses of the fMRI data used univariate voxel-based statistical techniques. At the individual subject level, general linear models (GLMs) were fit to the data using a set of boxcar functions for the conditions of interest convolved with a standard hemodynamic response function. It should be noted that all regressors of interest were scaled such that the modeled hemodynamic response had a range of −1 to 1 to facilitate interpretation of beta coefficients in terms of percent signal change and comparisons between beta coefficients. GLMs were run after concatenating all EPI runs with regressors of interest for each analysis described below in addition to movement estimates as additional regressors. A separate constant regressor was used for each run to allow for between run differences in means. Each group-level analysis, unless otherwise noted, was done using a mixed effects approach using AFNI’s 3dMEMA command where the average and variance (i.e., beta coefficient and *t*-statistic) at the individual participant level were used in the group analysis (Chen et al., 2012).

Unless otherwise specified, all results were corrected for multiple comparisons using family-wise error cluster size thresholding to a corrected *p*-value of less than 0.05 by setting the uncorrected *p*-value threshold to 0.001 and a cluster size of 15 voxels (Forman et al., 1995). Cluster size was determined using AFNI’s 3dClustsim, which allows for determination of cluster size using Monte Carlo simulations.

#### Strategy Contrast Analyses

Regressors for reading, self-explanation, and paraphrasing were included in the GLMs (and rereading for the two prior studies containing that strategy). In addition, the mind wandering and comprehension monitoring rating tasks were modeled with separate regressors. These regressors are not analyzed in this paper and were included only so that any variance associated with these regressors was not captured by strategy regressors. Group-level contrasts between the reading strategies could be performed with these regressors. These GLMs were used as the base model for the functional connectivity analyses to which PPI regressors were added.

In addition to being used in the PPI analyses, a group analysis collapsing across the three studies was run to provide a statistically powerful examination of the differences in activity levels for self-explanation and paraphrasing. Because of the large clusters of activity that would have been less informative at the uncorrected *p*-value of 0.001 (as used in all other analyses), an uncorrected *p*-value of 0.0001 was used along with a cluster size of 7 to achieve a family-wise error corrected *p*-value of 0.05.

#### PPI Analysis

**Figure 2:**
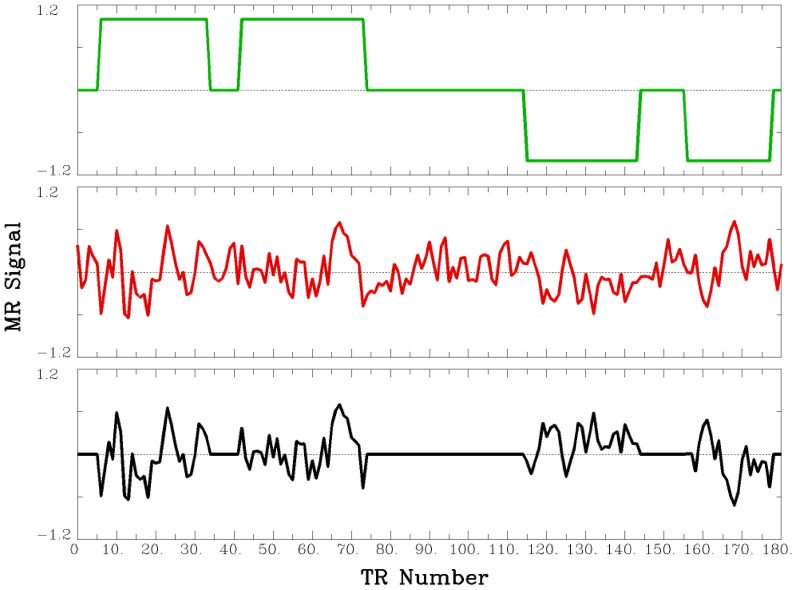
**Example of PPI regressors.** The top panel (green line) shows the coding of self-explanation (as 1) and paraphrasing (as −1) periods across 180 TRs. The middle panel (red line) shows the seed ROI time course as extracted from the group of voxels in the ROI after linear trends and the mean was removed, and the interaction (PPI) between these two lines (green and red) is shown by the black line in the bottom panel. The red and black lines were used as regressors in the PPI analyses.

This PPI analysis examined functional connectivity during the times when participants were performing the paraphrasing task as compared to times when they were performing the self-explanation task. For this analysis, the three seed ROIs in Table 1 were used. For each participant’s data, each ROI’s average time series was extracted and the time series was temporally filtered to remove linear trends and the mean was removed (shown in the middle part of Figure 2). The time series was deconvolved using a gamma hemodynamic response function to generate the time series of the underlying neuronal signal for the ROI. The PPI regressors for example data are shown in Figure 1. An interaction regressor was obtained by first coding each TR with a value of 1 for the self-explanation task and −1 for the paraphrasing task (top part of Figure 2), and then this task time course was multiplied element-by-element with the estimate of the underlying neuronal signal obtained through the deconvolution of the average time series for the seed ROI. Finally, the resulting vector obtained by the element-by-element multiplication was convolved with a gamma hemodynamic response function to obtain the interaction regressor (shown by the bottom part of Figure 2). The regressors for the average activity in the seed ROI and the resulting interaction regressor were both entered as additional regressors to the model already described above for the reading strategy contrasts. The regressor of interest for this analysis is the interaction regressor that accounts for activity in regions outside of the seed ROI over and above the effects of the task and the overall pattern of activity in the seed ROI (i.e., activity associated not with the seed ROI but only task-induced changes in correlation with the seed ROI).

#### Human Connectome Resting-State Data

The Human Connectome Project (Van Essen et al., 2012, 2013; Smith et al., 2013) released resting state connectivity analyses from a set of 468 participants. This dataset was used to get a qualitative sense of the brain networks involved in coherence building during self-explanation. To examine this data, the resting state correlation data with the mean grayordinate time series regressed out was used (Smith et al., 2013). Generally, any given gray matter region is at least weakly positively correlated with other gray matter regions. Regressing out the mean time series means that the net correlation across all regions is zero and some regions become anti-correlated (Smith et al., 2013). With this data, the peak coordinate from each ROIs determined by the PPI analyses were used as a seed for the resting state data, and the correlation map was thresholded at *Z* > 2.5. This process yielded figures showing regions that were positively correlated with each seed region and each region found in the PPI analysis in this paper.

## Results

### PPI Analyses

For each of the three ROIs that are consistently more active for self-explanation than for paraphrasing (see Table 1), a PPI analysis was run to identify regions that exhibited significantly changed functional connectivity with the seed in a contrast of self-explanation vs. paraphrasing blocks. The purpose of this type of analysis is to uncover networks that play a role in effective reading strategies but may not change in overall activity and thus are not detected by a traditional univariate overall activity contrast.

**Figure 3:**
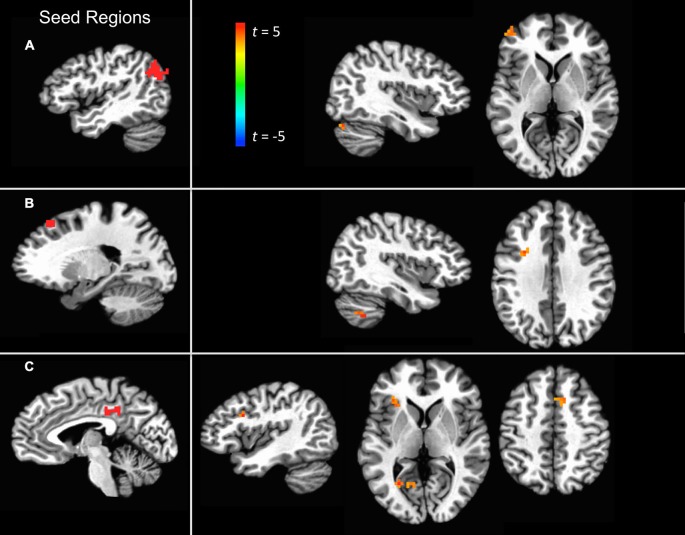
**Significant clusters showing a PPI effect with the three seed regions: (A) the angular gyrus seed region, (B) the dSFG seed region, and (C) the PCC seed region**.

For the angular gyrus ROI seed, the cerebellum and the left anterior middle frontal gyrus portion of the dorsolateral prefrontal cortex (DLPFC) exhibited increased connectivity with this region during self-explanation (see part 1 of Table 2; Figure 3A). For the dSFG ROI seed, the cerebellum and left IFJ both exhibited increased functional connectivity with this seed region (see part 2 of Table 2; Figure 3B). For the PCC ROI seed, the left dorsal inferior frontal gyrus (dIFG), left lingual gyrus, right SMA, and the left anterior insular cortex (AIC) all exhibited increased functional connectivity with the seed region (see part 3 of Table 2; Figure 3C). Notably, no region in any of these analyses showed greater functional connectivity with any of the seed regions for paraphrasing relative to self-explanation, which is consistent with the interpretation that self-explanation is a complex process that recruits additional regions and networks to implement the inferencing strategies needed to form a coherent situation model.

**Table 2:** **Regions showing a significant PPI effect for the change between performing the self-explanation and paraphrasing strategies for each of the three seed regions**.

Regions	BA	*x*	*y*	*z*	Peak *t*	Size (mm^3^)
*Angular ROI seed*
R cerebellum—VIIa crus		41	−70	−25	4.92	756
L middle frontal g (DLPFC)	10, 46	−40	50	5	4.08	675
*Frontal ROI seed*
R cerebellum—VIIa crus		38	−52	−43	4.60	405
L IFJ	6, 9	−28	8	32	4.41	513
*PCC ROI seed*
L dorsal inferior frontal g	9, 46	−40	11	26	4.63	594
L lingual g	18, 19	−10	−58	2	4.05	621
R SMA	6, 32	11	14	44	4.04	513
L AIC	13, 45	−31	26	5	4.19	405

**Figure 4:**
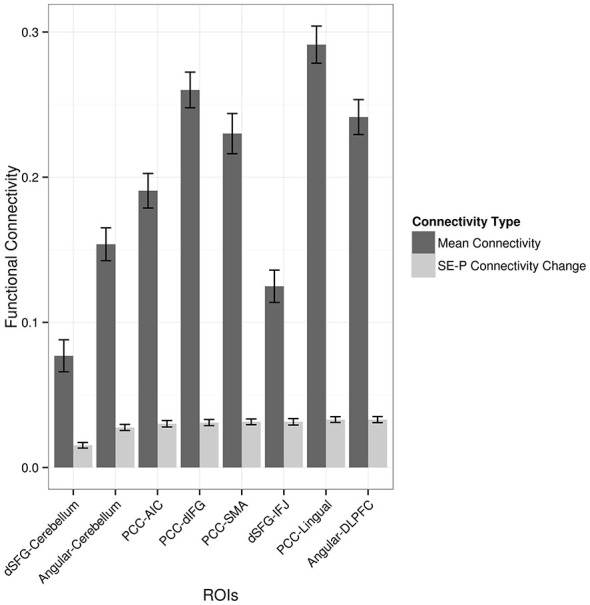
**Average functional connectivity throughout the task and PPI increases in connectivity when self-explaining compared to paraphrasing.** Values shown are correlation values for the seed region regressor and the PPI regressor. ROIs are given as pairs with the seed region followed by the region from Table 2.

Each of the regions showing an increase in functional connectivity with the seed regions was examined to see whether overall functional connectivity was related to the extent to which functional connectivity was modulated by the paraphrasing and self-explanation strategies. For example, did modulation only occur in areas that were generally strongly connected in general with the seed region across all strategies? Figure 4 shows both mean functional connectivity from the seed time course regressor and the SE-P connectivity change in each area from the PPI regressor. Overall mean and SE-P connectivity change were not strongly associated; self-explanation appeared to strengthen both stronger and weaker connections.

### Self-Explanation Vs. Paraphrasing Comparison

**Figure 5:**
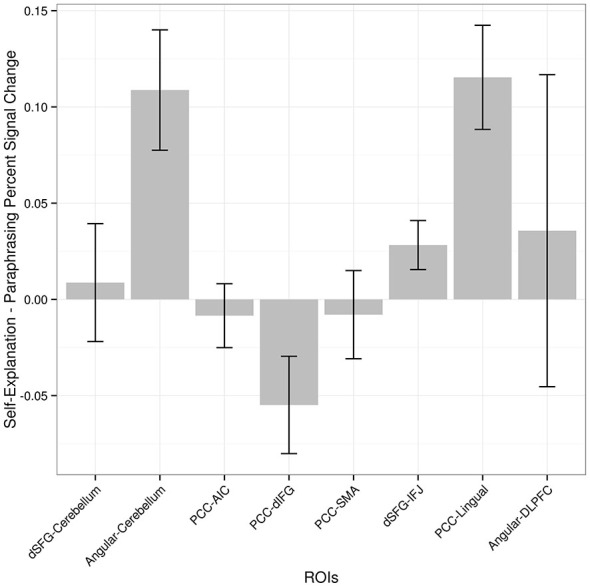
**Percent signal change (self-explanation minus paraphrasing) in each region found in the PPI analysis.** Regions are ordered as in Figure 3 to allow for comparison between size of PPI effect and difference in activation for self-explanation and paraphrasing. The pairing of seed region to target region is maintained in this figure to match Figure 3 even though percent signal change in this graph does not depend on the seed region.

Based on the functional connectivity data alone it is not clear whether the identified regions increase in functional connectivity alone or also have accompanying increases in overall activation that did not meet the statistical threshold necessary to show up in the prior conjunction analysis that yielded the three seed regions. In order to examine this possibility, the percent signal change from paraphrasing to self-explanation of each cluster of active voxels from the PPI analysis is plotted in Figure 5. These values were obtained from a GLM including all of the regressors for strategies as described earlier but leaving out any PPI-related regressors. Three regions including one cerebellum cluster, the middle frontal gyrus cluster, and the lingual gyrus cluster all show increases in activity during self-explanation as well as increases in functional connectivity during this strategy. The dIFG cluster actually shows greater activity during paraphrasing even though it is higher in functional connectivity with the PCC seed during self-explanation. All other regions do not show statistically significant differences in overall activity during self-explanation.

**Figure 6:**
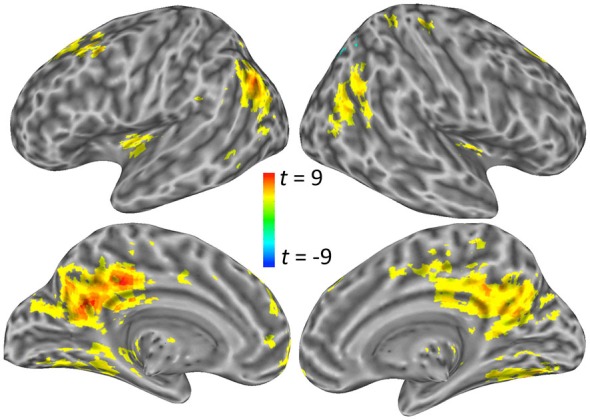
**Significantly active clusters for the self-explanation— paraphrasing contrast**.

To further explore these increases in activity, a contrast between self-explanation and paraphrasing was conducted without any functional connectivity regressors. Significant clusters of activation are shown in Figure 6 and in Table 3. The largest peaks correspond to the PCC, angular gyrus, and dSFG seed regions found in the prior conjunction analysis (Moss et al., 2013). However, a number of additional regions were found including the lingual gyrus and cerebellum regions as expected based on the data in Figure 5. Right hemisphere homologs of the left hemisphere seed regions were found including right angular gyrus, right dSFG, and right PCC (activation that was hard to distinguish from left hemisphere PCC peaks in this region). Notably, medial prefrontal regions were found including a region of the vmPFC and dmPFC normally associated with theory-of-mind processing. Also, activity in the parahippocamal gyrus and hippocampus showed increases with self-explanation. These results suggest a bilateral network of brain regions with more activity in the left hemisphere that supports effective strategic reading comprehension. Some of these regions were also found in one of the prior papers using some of these data (Moss et al., 2011).

**Table 3:** **Significant peaks of activation for the self-explanation—paraphrasing contrast**.

Regions	BA	*x*	*y*	*z*	Peak *t*	Size (mm^3^)
*Frontal Cortex*
L middle frontal g	6, 8	−26	14	48	7.38	7,506
L superior srontal g (frontal ROI)	8	−17	26	48	6.89	−
R superior frontal g	8	23	26	45	5.15	540
L vmPFC	10	−4	59	5	5.91	2,484
L posterior insula	13	−34	−10	2	5.67	2,241
R posterior insula	13	40	−8	2	6.00	729
L dmPFC	9	−4	43	23	5.48	702
B middle cingulate g	24	5	−5	39	4.99	513
*Parietal Cortex*
L PCC (PCC ROI)	23, 31	−5	−32	36	10.00	41,013
L precuneus	23, 29, 30	−5	−50	18	9.87	−
L precuneus	7, 31	−2	−71	30	8.12	−
L angular g (angular ROI)	39	−41	−65	36	8.23	10,665
R angular g	39	50	−61	26	7.30	5,022
R supramarginal g	40	−50	−32	27	4.97	270
R postcentral g	3	29	−32	51	5.81	378
R postcentral g	5, 7	23	−44	54	5.59	324
R precuneus	5, 7	8	−41	51	5.13	297
L superior parietal	7	−23	−62	42	−4.80	243
R superior parietal		26	−62	45	−4.86	243
*Temporal Cortex*
R parahippocampal g	19, 36, 37	26	−44	−4	6.12	378
L hippocampus	28, 35	−20	−20	−10	5.20	243
L middle temporal g	20, 37	−53	−41	−7	4.84	243
*Occipital Cortex*
R lingual g	18, 19	20	68	−7	7.13	4,428
*Subcortical/Cerebellum*
L thalamus		−5	−17	21	5.33	216
R thalamus		2	−17	9	4.99	405
R thalamus (pulvinar)		14	−32	9	4.91	270
R putamen		29	−5	3	5.55	324
L caudate		−5	−5	15	4.70	243
R cerebellum—VIIa crus I		26	−77	−22	4.75	351

*A dash in the size column means that the peak was contained within the preceding larger cluster of activation*.

### Resting-State Connectivity

These PPI and mean activity contrast results provide a characterization of processing during a coherence-building reading strategy. However, these results do not provide much insight into the networks that the three seed regions may be tapping into more generally. For example, with what regions is the DLPFC region (showing a PPI-related increase with the angular gyrus seed region) communicating on a routine basis? One source of data to address this kind of questions comes from resting-state fMRI data.

**Figure 7:**
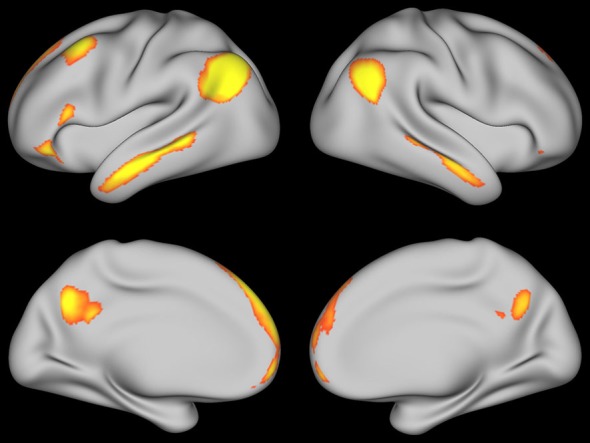
**Resting state map generated using the angular gyrus seed region thresholded at *Z* > 2.5**.

**Figure 8:**
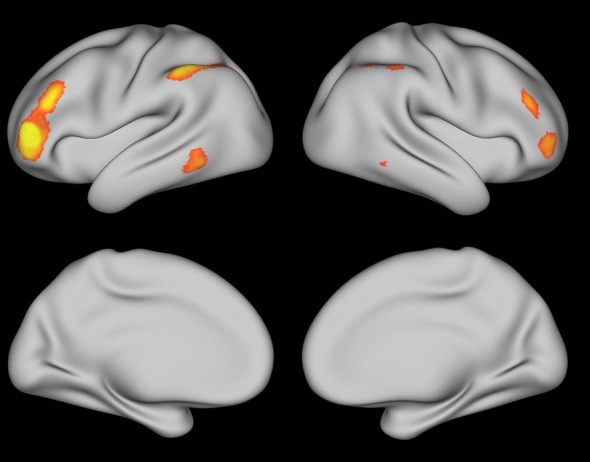
**Resting state map generated using the DLPFC region from Table 1 as the seed region thresholded at *Z* > 2.5**.

Resting-state data from the Human Connectome Project (Van Essen et al., 2012, 2013; Smith et al., 2013) were used to get a qualitative sense of the brain networks involved in coherence building during self-explanation. With this data, the peak coordinate from each region in Tables 1, 2 was used as a seed for the resting state data. Figures 7, 8 show regions that were positively correlated with each seed region as well as each region found in the PPI analysis in this paper. Figure 7 was generated using the angular gyrus ROI from Table 1; Figure 8 was generated using the DLPFC ROI from Table 2. In each of these regions, increasing correlations between the depicted regions and the seed region are shown from red to yellow.

**Figure 9:**
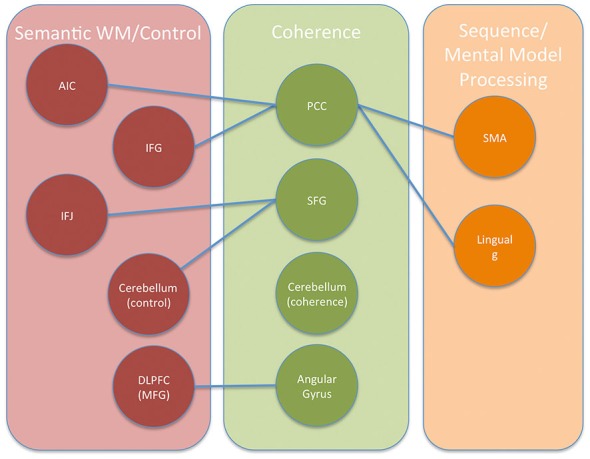
**Conceptual diagram showing the regions and networks involved in building coherent representations of expository text using self-explanation.** Three networks are depicted based on patterns of connectivity observed from the HCP functional connectivity results and the PPI results. Connections between networks are shown with lines where each line is based on the presence of a change in connectivity found in the PPI results.

These two figures were selected from the entire set because they were representative and many of these ROIs yielded very similar maps of resting state activity. For Figure 7, generated from the angular ROI, the PCC and SFG seed regions in Table 1 yield a similar map as does the cerebellum region found with the angular gyrus seed region in Table 2. The fact that these regions all show similar patterns of resting-state connectivity supports the characterization of these regions as a network supporting coherence-building as is shown conceptually in Figure 9. For the DLPFC seed region shown in Figure 8, the IFG, IFJ, middle frontal gyrus, and the cerebellum region associated with the PCC seed from Table 2 all yield a similar resting-state network. This network is identified as a semantic working-memory control network in Figure 9.

Finally, the lingual gyrus and SMA regions from Table 3 yielded distinct maps showing a diffuse pattern of connectivity to many regions including weak connections to each other. The SMA region showed this diffuse pattern of activity primarily over motor, premotor, somatosensory, and some higher-order visual processing regions along the occipital-parietal boundary. The lingual gyrus showed a diffuse pattern of connectivity over most of occipital cortex, medial prefrontal regions, and some motor/somatosensory regions along the central sulcus. These two regions are identified as potentially part of a network involved in sequential processing in Figure 9.

## Discussion

The current study examined the brain regions involved in constructing explanations to aid comprehension by examining regions that increased in functional connectivity when engaging in a reading strategy that emphasized explaining material to oneself. Seed regions for the PPI analyses included the left angular gyrus, left dorsal SFG, and left PCC regions (see Coherence areas in Figure 9). These seed regions were selected because they were more active in self-explanation than in another reading strategy, paraphrasing, that also engaged cognitive control regions but does not focus on constructing inferences to make explanations (Moss et al., 2013). Each of these seed regions was found to increase in functional connectivity with a small unique set of regions (see Table 2; Figure 9). Two additional analyses were conducted to further characterize these regions and the broader networks in which they participate.

In the first set of additional analyses, these connected regions were examined to determine whether they were more active in self-explanation than in paraphrasing at all by examining changes in activation levels during the performance of these strategies. The left lingual gyrus and a region in the cerebellum both showed increases in activation with self-explanation along with increased functional connectivity with the coherence building regions. The dIFG actually showed a decrease in overall activation levels with self-explanation although it showed an increase in functional connectivity with the PCC ROI during self-explanation. As discussed further below, this region has been theorized to provide access to the products of semantic memory retrieval (Anderson et al., 2003). Increased communication between the PCC and this region might reflect the incorporation of new semantic information into a situation model. Paraphrasing may actually involve more memory retrieval overall, but this retrieval may be more for finding synonyms for words contained in the text and so it would not be incorporated into the situation model.

Regions beyond the three original seed ROIs were also identified in the contrast of the self-explanation to paraphrasing strategy including right hemisphere homologs of the left hemisphere ROIs. Interestingly, other regions of the “mentalizing” network (Mar, 2011; Spunt and Lieberman, 2012) including dmPFC and vmPFC were identified as more active during self-explanation. These regions are noteworthy because the dmPFC has been argued to underlie theory-of-mind processing in social situations and narrative text (Ferstl and von Cramon, 2001, 2002; Spunt and Lieberman, 2012), but the texts used in the current task were expository biology and physics texts that do not lend themselves to theory-of-mind processes. One possible unifying explanation is that theory-of-mind processes entail positing hidden goals and relating actions of others to these hidden goals. In expository text, many of the described entities have particular functions and one might think of these entities as “wanting” to perform these functions. In other words, there may be common cognitive processes that underlie our ability to explain the interactions of entities in some expository biology text (e.g., a text describing the process of cell mitosis) and that also underlie our ability to explain why someone is performing some action in a social situation or story. vmPFC is often associated with assessing value in a decision making process (e.g., Hare et al., 2009), and it could be that during self-explanation one has to decide on the value of one best inference amongst a set of possible inferences that could be drawn. Regardless of the reason, the finding that these same regions activate for expository text is noteworthy and highlights the common functions being played by these regions across a variety of tasks that require constructing a coherent representation.

In the second set of additional analyses, resting state data from the Human Connectome Project were examined to more broadly characterize the networks in which these PPI-identified regions sit. Two primary networks were identified. The first network is similar to what has been termed the mentalizing or default mode network (Raichle et al., 2001; Buckner et al., 2008; Mar, 2011). While many of the nodes in the mentalizing/default mode network seem to also appear related to coherence building, the medial prefrontal cortex regions exhibit a smaller response in our strategic reading paradigm than in narrative comprehension or social cognition paradigms. Therefore, we draw the distinction between the mentalizing network and a subset of this network that we have termed a coherence-building network. In addition, the self-explanation vs. paraphrasing contrast also included regions such as the anterior IFG as well as middle and anterior temporal lobes (see Figure 6). This larger network has been identified as a general semantic network based on a meta-analysis of many tasks involving retrieval and manipulation of semantic memory (Binder et al., 2009).

The second network included regions such as DLPFC, AIC, IFJ, and dIFG as shown in Figure 9. On the basis of the rs-fMRI data shown in Figure 8, additional regions such as dorsal angular gyrus, ACC, pre-SMA, and parts of the middle temporal lobe might be included in this network. Many of these regions have been previously identified as regions showing decreases in activity with increased practice (Chein and Schneider, 2005) and have been termed a cognitive control network (Cole and Schneider, 2007). However, the subset of the control network identified in the PPI analysis might be considered to be those parts of the control network most specialized to the controlled manipulation of semantic representations.

Interestingly, two regions identified in the PPI analyses, the SMA and lingual gyrus regions, did not fall into these other two networks. These two regions were specifically connected to the PCC in the PPI analyses, and they may reflect the recruitment of regions for representing sequential sequences of action and the use of visuospatial mental models as discussed further below.

The role of these networks can be understood with respect to cognitive theories of comprehension. In the sections that follow, we outline possible roles for these networks in the process of comprehension as well as potential functions for many of the prominent regions in these networks drawing both on comprehension theory and the neuroimaging literature.

### Construction-Integration Theory of Comprehension

According to Kintsch’s (1998) broadly used construction-integration theory of comprehension, there are three levels of text representation. First, is the surface representation, which involves the words actually contained in the sentence (i.e., the exact wording). Second, during the construction phase, this surface representation is converted to propositions during reading, and these propositions from the text are referred to as the textbase representation. Also during the construction phase, additional knowledge from semantic memory is activated based on similarities in the propositions in the textbase. Third, this elaborated textbase with activated propositions from long-term memory is the situation model. The situation model then is processed in the integration phased during which a parallel constraint satisfaction process operates to help resolve ambiguities and conflicts in the representation. The resulting situation model can be more or less coherent depending on how well connected propositions were in the original text (i.e., text cohesion) and how well prior knowledge elaborated on the text base to form a coherent situation model.

These processes in the Construction Integration model highlight the importance of both text cohesion and prior knowledge in comprehension, and indeed these factors have been shown to affect how much is learned from text (McNamara and Kintsch, 1996; McNamara et al., 1996). In addition to these fairly automatic comprehension processes, controlled inference making can be used to further enhance a situation model’s coherence. These controlled inferences are exactly the types of processes taught during self-explanation (McNamara, 2004). Construction of a well-connected set of propositions including relevant inferences allows for a situation model that can be used to explain the system of interacting elements often being described in expository text.

These controlled inference processes may not be as necessary for social situations or narrative text because the comprehender is often an adult with multiple decades worth of related prior knowledge concerning likely motives and actions that can be attributed to the protagonist and associated characters. In these cases, it is likely that the automatic nature of the construction-integration process produces coherent representations with little conscious effort. This difference in narrative and expository text with respect to the prior knowledge of the comprehender may help to understand why a common network is active for all kinds of comprehension, but that when more control is needed, such as in self-explanation of expository text, that additional brain networks increase in functional connectivity to this common coherence building comprehension network.

### Coherence-Building Network

Regions of this coherence-building network have been observed in studies of sentence and discourse comprehension including a recent meta-ananlysis of such studies (Ferstl et al., 2008). In particular, regions including the angular gyrus, middle temporal gyrus, anterior temporal cortex, vmPFC, dmPFC, anterior IFG, precuneus, and PCC were identified in this meta-analysis based on studies that examined coherence building by contrasting coherent and incoherent text (Fletcher et al., 1995; Maguire et al., 1999; Ferstl and von Cramon, 2001, 2002; Xu et al., 2005; Ferstl et al., 2008). A similar set of regions has been identified in the social neuroscience literature as pertaining to mentalizing or thinking about one’s own or another’s thought processes (Mar, 2011; Spunt and Lieberman, 2012). Finally, a larger network of regions that includes this coherence network as a subset has been identified in a meta-analysis of semantic memory and manipulation of semantic representations (Binder et al., 2009). There are likely multiple functions included in this network of brain regions, and the current results provide some additional insight into the connectivity of regions of this network to other regions. Regions such as dmPFC and vmPFC respond to a smaller degree in the current strategic reading paradigm, likely reflecting the fact that their core processes have more to do with theory-of-mind processing than coherence building.

#### The Role of the PCC

The PCC was found to increase in functional connectivity with the AIC, dIFG, SMA, and lingual gyrus. A recent review discussed the role that the PCC plays in regulating internal/external focus of attention as well as the scope of attention through its connection to a dorsal attention network and a fronto-parietal control network (Leech and Sharp, 2014). The PCC’s increased functional connectivity with the AIC and dIFG seem likely related to its role in regulating portions of the control network. Similarly, increased resting-state connectivity of the PCC with anterior medial PFC was found to be related to individual differences in maintaining task focus during reading (Smallwood et al., 2013). Another related proposal is that the PCC is involved in a multi-modal episodic buffer for maintaining episodic and event information (Baddeley et al., 2010; Newman et al., 2013). The maintenance of event information may help to explain the increased connectivity to SMA and the lingual gyrus which may be related to maintaining sequential order in mental representations as discussed below.

#### The Role of the Angular Gyrus

The angular gyrus was found to increase in functional connectivity with a DLPFC region and a region of the cerebellum during self-explanation. This ventral portion of the angular gyrus has been most closely associated with the top-down allocation of attention to semantic memory including the use of semantic constraints in self-referential processing (Seghier, 2013). It has been shown to be a key hub between anterior prefrontal regions and semantic representations in the temporal cortex (Saur et al., 2010). As discussed below, the DLPFC region has been associated with the manipulation of relations amongst elements in working memory (Blumenfeld and Ranganath, 2006). Forming inferences based on prior knowledge involves the manipulation or creation of relations amongst elements in working memory based on semantic knowledge. The angular gyrus therefore seems likely to be involved in this aspect of coherence building. Furthermore, the connection to the cerebellum may mean that the angular gyrus is sensitive to error-related signals in coherence. The cerebellum has traditionally been associated with error-correction in motor control, but recent evidence of distinctly cognitive control related circuits have led to a theory in which the cerebellum provides error signals for use in non-motor cognitive computations (Ito, 2008). Under such a theory, the cerebellum would provide feedback on differences between predicted consequences of a cognitive process and the actual outcome. Such an error signal might be used to aid in top-down selection of which amongst multiple inferences might improve coherence in a situation model.

#### The Role of the dSFG

Finally, the dSFG region of this network showed increased connectivity with the IFJ and a region of the cerebellum showing connectivity with the control network. These are both regions of the cognitive control network (Chein and Schneider, 2005; Cole and Schneider, 2007), and this connectivity could be plausibly explained as a mechanism by which comprehension-related task goals could help to modulate activity in these regions. In particular, the IFJ is particularly important for top-down modulation of attention in working memory as discussed below (Brass et al., 2005; Gazzaley and Nobre, 2012).

#### Lateralization in the Network

Other noteworthy aspects of the results pertaining to this network is that activation was more left-lateralized during self-explanation of expository text and the role of this network in default-mode activity. While there has been debate about whether the lateralization of discourse comprehension and inference generation, the majority of evidence seems to point to a bilateral comprehension network (Ferstl et al., 2008; Saur et al., 2010). However, there has been some evidence that the types of inferences might affect lateralization by research showing that intention-related inferences related to theory-of-mind and inferences about the physical consequences of actions might differ in lateralization with the causal inferences about physical actions being left lateralized (Mason and Just, 2011). Therefore, the left lateralization seen here may be because of the expository material.

Finally, this same network has substantial overlap with the default mode network. The default mode network is engaged when individuals are not conducting a task-related goal, and it is likely that this network is involved in mentalizing about one’s thoughts and plans (e.g., Mar, 2011). This type of thought is likely to involve similar manipulation of complex propositional information like that found in situation model representations that are the result of comprehension. This relationship may also explain why mind wandering is so detrimental to reading comprehension (Smallwood et al., 2007, 2013; Moss et al., 2013).

In summary, this network may be engaged in the routine and automatic components of the construction-integration process that results in a situation model where relevant prior knowledge is easily activated and integrated to form a coherent representation. This process is likely common to many kinds of comprehension including those in narrative text and social situations, but with low cohesion expository text and relatively little relevant prior knowledge, additional comprehension processes may be needed that include carefully controlled manipulation of semantic knowledge.

### Cognitive Control and Semantic Manipulation Network

The second primary network that appears to be engaged in coherence building is the cognitive control network. Components of this network such as the DLPFC, ACC, AIC, dPMC, PPC, ACC/pSMA, and the cerebellum have been implicated in cognitive control because of their consistent decrease in activity with increasing practice at a task (Chein and Schneider, 2005). These regions have also been shown to have a high degree of functional connectivity (Cole and Schneider, 2007). Discussion here will focus on components of this network specifically identified in the present results and their role in comprehension.

#### The Role of the DLPFC

First, the DLPFC region identified in the middle frontal gyrus has been associated primarily with the manipulation of relations within working memory (Blumenfeld and Ranganath, 2006). The anterior IFG has been associated with retrieval and maintenance of semantic information within working memory, but the middle frontal gyrus seems to be engaged only when manipulation of the relations between retrieved items are required (Blumenfeld and Ranganath, 2006). Because both self-explanation and paraphrasing require the retrieval of semantic information, the anterior IFG is likely engaged to an equivalent degree for both reading strategies. However, only self-explanation emphasizes inference construction. Inference construction requires the creation and manipulation of relations between different propositions, and therefore the middle frontal gyrus is more active during self-explanation than during paraphrasing. The increased functional connectivity of this DLPFC region with the angular gyrus may occur because of the use of constraining semantic information maintained in the angular gyrus that is necessary to create the appropriate relation between items in semantic working memory.

#### The Role of the IFJ

A second region of the control network showing changes in functional connectivity is the IFJ. The IFJ has been posited to control the top-down modulation of attention within working memory to task-relevant representations (Brass et al., 2005; Gazzaley and Nobre, 2012). Such modulation would be needed to activate the relevant information in working memory as relations (i.e., inferences) are being formed between specific items in working memory. The IFJ increased in functional connectivity with the dSFG region which also showed an increase in a region of the cerebellum associated with the control network via the pattern of resting-state connectivity that region of the cerebellum showed. It may be that the cerebellum is relaying error-related signals pertaining to control and the dSFG is helping to maintain task-related goals that help to modulate IFJ control of working memory.

#### The Roles of the AIC and dIFG

Both the AIC and dorsal IFG showed increases in connectivity with the PCC during self-explanation, and are argued to be part of semantic control functions in coherence building. The dorsal portion of the IFG is close to a region that has been called a declarative memory buffer in the ACT-R cognitive architecture (Anderson et al., 2003). According to that theory, this buffer holds relevant propositional representations retrieved from declarative memory so that they can be accessed and used to guide cognition. Selecting the next cognitive action based on the contents of long-term memory is a relatively common task, and therefore it makes sense that this region would be a part of the brain’s cognitive control network. The PCC may be using information about relevant long-term memory to modulate internally-directed attention to incorporate this information into the situation model being developed. The decrease in level of activity in this region for self-explanation relative to paraphrasing may reflect a decrease in the use of memory retrieval relative to paraphrasing because memory retrieval may be operating constantly during paraphrasing to find synonyms for words in memory to use in paraphrases. However, paraphrasing retrieval activity is relatively unrelated to improving the situation model.

The AIC is thought to be part of a salience network with strong connectivity to the ACC and other cognitive control regions (Menon and Uddin, 2010). The functional connectivity between these three regions may be a mechanism that allows the contents of the current situation model and retrieved memories to influence the salient information guiding other components of the control system in the formation of new inferences.

To summarize, the portion of the cognitive control network observed to increase in functional connectivity seems to be those nodes most associated with the manipulation and attention to relations between semantic information in working memory. Indeed, a region of the posterior parietal cortex in the cognitive control network associated with externally-directed top-down attention was the sole region showing more activation in paraphrasing than in self-explanation in Figure 6; Table 1. This semantic control network appears to interact with the coherence building network described previously when effortful construction of new inferences needs to occur to create a coherent situation model.

### Sequential Mental Model Regions

The last set of regions to be discussed are the SMA and lingual gyrus regions found to increase in functional connectivity to the PCC seed region during self-explanation. The SMA and lingual gyrus show broad patterns of diffuse resting state activity that may be indicative of their role in many networks that rely on the types of processing done in these regions. The SMA region has been shown to be associated with the learning of uncued sequences of motor actions (Deiber et al., 1999) as well as concepts involving space and time (Beudel et al., 2009), and it has been seen in other comprehension studies where it was hypothesized that it is involved in the sequential representation of events in narrative comprehension (AbdulSabur et al., 2014). The lingual gyrus is often associated with visuospatial processing, but it has also been found to be active during logical deduction tasks where participants do not have familiar semantic knowledge to use in deduction (Goel et al., 2004). Goel et al. (2004) hypothesized that in the absence of other representations that can be used in logical inference, that people build visuo-spatial mental models (Johnson-Laird and Byrne, 1991) that are used in reasoning. The integration of sequences with visuo-spatial mental models is at least a plausible explanation for the role of these regions in expository text comprehension especially when considering that the texts used here described the causal interaction of different elements in space and time for the various systems described in the texts (e.g., pulley systems, structure and function of DNA).

## Conclusion

In conclusion, the analysis of changes in functional connectivity during different reading strategies along with the investigation of overall activation differences and resting-state functional connectivity provides some insight into the potential neural mechanisms underlying the construction of coherent representations that are the products of effective comprehension. A coherence-building network involving the construction of situation models was identified that corresponds to networks also observed in mentalizing. In addition, portions of the brain’s cognitive control network that appear to specialize in the controlled manipulation of semantic information may underlie the ability to construct new inferences to help form coherent situations in challenging situations with low cohesions expository text and little relevant prior knowledge. Mappings between current cognitive comprehension theory and these brain networks provide some basis for new hypotheses about the function of the nodes of these respective networks that can be used to formulate future research objectives to better understand the component functions and the interactions of these regions.

## Conflict of Interest Statement

The authors declare that the research was conducted in the absence of any commercial or financial relationships that could be construed as a potential conflict of interest.

## Abbreviations

ACC: anterior cingulate cortex
AIC: anterior insular cortex
dIFG: dorsal inferior frontal gyrus
DLPFC: dorsolateral prefrontal cortex
dmPFC: dorsomedial prefrontal cortex
dSFG: dorsal superior frontal gyrus
DTI: diffusion tensor imaging
fMRI: functional magnetic resonance imaging
GLM: general linear model
IFG: inferior frontal grysu
IFJ: inferior frontal junction
PCC: posterior cingulate cortex
PPI: psychophysiological interaction
ROI: region of interest
rs-fMRI: resting-state functional magnetic resonance imaging
SERT: self-explanation reading training
SMA: supplementary motor area
vmPFC: ventromedial prefrontal cortex.
